# The liver-resident immune cell repertoire - A boon or a bane during machine perfusion?

**DOI:** 10.3389/fimmu.2022.982018

**Published:** 2022-10-13

**Authors:** M. Fodor, S. Salcher, H. Gottschling, A. Mair, M. Blumer, S. Sopper, S. Ebner, A. Pircher, R. Oberhuber, D. Wolf, S. Schneeberger, T. Hautz

**Affiliations:** ^1^ Department of Visceral, Transplant and Thoracic Surgery, Center of Operative Medicine, organLife Laboratory, Medical University of Innsbruck, Innsbruck, Austria; ^2^ Department of Visceral, Transplant and Thoracic Surgery, Daniel Swarovski Research Laboratory, Medical University of Innsbruck, Innsbruck, Austria; ^3^ Department of Internal Medicine V, Hematology and Oncology, Comprehensive Cancer Center Innsbruck (CCCI), Medical University Innsbruck (MUI), Innsbruck, Austria; ^4^ Department of Anatomy and Embryology, Medical University of Innsbruck, Innsbruck, Austria

**Keywords:** liver transplantation, machine perfusion, liver-resident immune cells, immune activation, innate immunity, adaptive immunity, ischemia reperfusion injury

## Abstract

The liver has been proposed as an important “immune organ” of the body, as it is critically involved in a variety of specific and unique immune tasks. It contains a huge resident immune cell repertoire, which determines the balance between tolerance and inflammation in the hepatic microenvironment. Liver-resident immune cells, populating the sinusoids and the space of Disse, include professional antigen-presenting cells, myeloid cells, as well as innate and adaptive lymphoid cell populations. Machine perfusion (MP) has emerged as an innovative technology to preserve organs *ex vivo* while testing for organ quality and function prior to transplantation. As for the liver, hypothermic and normothermic MP techniques have successfully been implemented in clinically routine, especially for the use of marginal donor livers. Although there is evidence that ischemia reperfusion injury-associated inflammation is reduced in machine-perfused livers, little is known whether MP impacts the quantity, activation state and function of the hepatic immune-cell repertoire, and how this affects the inflammatory milieu during MP. At this point, it remains even speculative if liver-resident immune cells primarily exert a pro-inflammatory and hence destructive effect on machine-perfused organs, or in part may be essential to induce liver regeneration and counteract liver damage. This review discusses the role of hepatic immune cell subtypes during inflammatory conditions and ischemia reperfusion injury in the context of liver transplantation. We further highlight the possible impact of MP on the modification of the immune cell repertoire and its potential for future applications and immune modulation of the liver.

## Introduction

Liver transplantation (LT) still remains the only treatment option for a variety of liver diseases eventually resulting in end-stage organ failure. Extended criteria donors (ECD) are increasingly used for transplantation to meet the high demand of organs. However, this poses a risk of early allograft dysfunction (EAD), primary non-function (PNF) and biliary complications [1-6]. Moreover, ECD livers are more susceptible to ischemia reperfusion injury (IRI), compared to standard criteria donor grafts.

While hypothermic conditions reduce cellular activity and metabolism during organ ischemia, accumulated toxins and reactive oxygen species (ROS) are released upon reperfusion, which initiates pro-inflammatory cascades, activates immune cells, releases damage associated molecular patterns and ultimately results in apoptosis and tissue necrosis (1–3). To limit organ damage during organ preservation, machine perfusion (MP) has emerged as an alternative to static cold storage (SCS). Normothermic machine perfusion (NMP) keeps a liver *ex vivo* in a complete functional state, close-to physiologic conditions and allows for comprehensive graft viability assessment before transplantation (4–9). An improved metabolic function, reduced expression of key markers of IRI and decreased activation of the immune response of NMP livers, compared to SCS livers, was previously demonstrated (2, 3).

**Figure 1:**
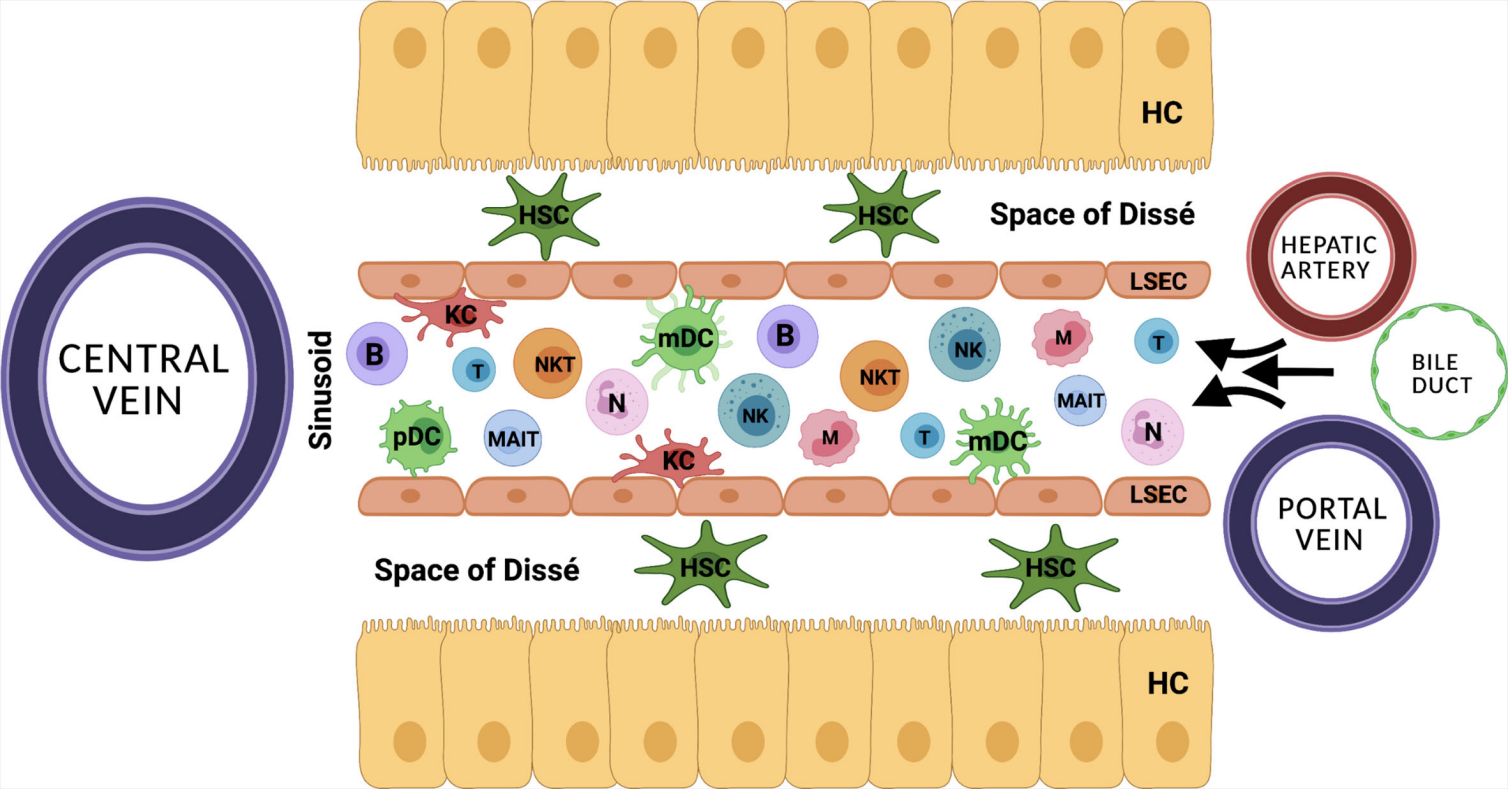
The distinctive anatomical vascular system allows for continuous exchange of immunological information. HC, hepatocyte; HSC, hepatic stellate cell; KC, Kupffer cell; B, B cell; T, T cell, NK, natural killer cell; NKT, natural killer T cell; N, neutrophil; MAIT, mucosa associated invariant T cell; mDC, myeloid dendritic cell; pDC, plasmacytoid dendritic cell; LSEC, liver sinusoidal endothelial cell.

The liver is essentially involved in balancing the innate and the adaptive immune system. Its anatomic position and distinctive vascular system allow for its unique ability to continuously exchange immunological information (**Figure 1**) (10, 11). Upon inflammation, the innate immunity including the complement system, pre-formed antibodies, as well as hepatic natural killer (NK) cells, macrophages and neutrophils, induces the inflammatory cascade and further initiates the adaptive immune response. Central to the hepatic adaptive immune system are T and B lymphocytes, which are able to recognize and reply to pathogens in an antigen-specific way, while natural killer T (NKT) cells function as a bridge between innate and adaptive immunity (12–15). To date, there is little evidence whether MP alters the quantity, activation state and function of hepatic immune-cells (16). The migration of donor passenger T cells from the donor liver allograft into recipient circulation has been demonstrated prior to the clinical use of NMP (17). A study by Jassem et al. reports an anti-inflammatory effect of NMP of donor livers and the promotion of liver regeneration (2). Recently, changes of the intrinsic immune profile of donor livers during NMP were confirmed. Specifically, it was suggested that liver-resident T cells and neutrophils are mobilized and released into the perfusate during NMP (18). Moreover, hypothermic oxygenated perfusion (HOPE) impressively reduced the number of liver-resident T cells and decreased cytokine levels, resulting in downregulation of the immune system and thereby preventing rejection and cholangiopathy after LT (19, 20). At this point it also remains speculative if liver-resident immune cells primarily exert a pro-inflammatory effect on the machine perfused organ, or in part may be essential to induce liver regeneration and counteract liver damage.

This review summarizes the function of various immune cell populations during hepatic immune responses with particular attention to inflammatory conditions in the context of LT. It discusses the potential role of the hepatic immune cell microenvironment during MP and if active modification of the immune cell repertoire may be advantageous during MP.

## Neutrophils are key players of immune cell activation as well as regeneration

**Figure 2:**
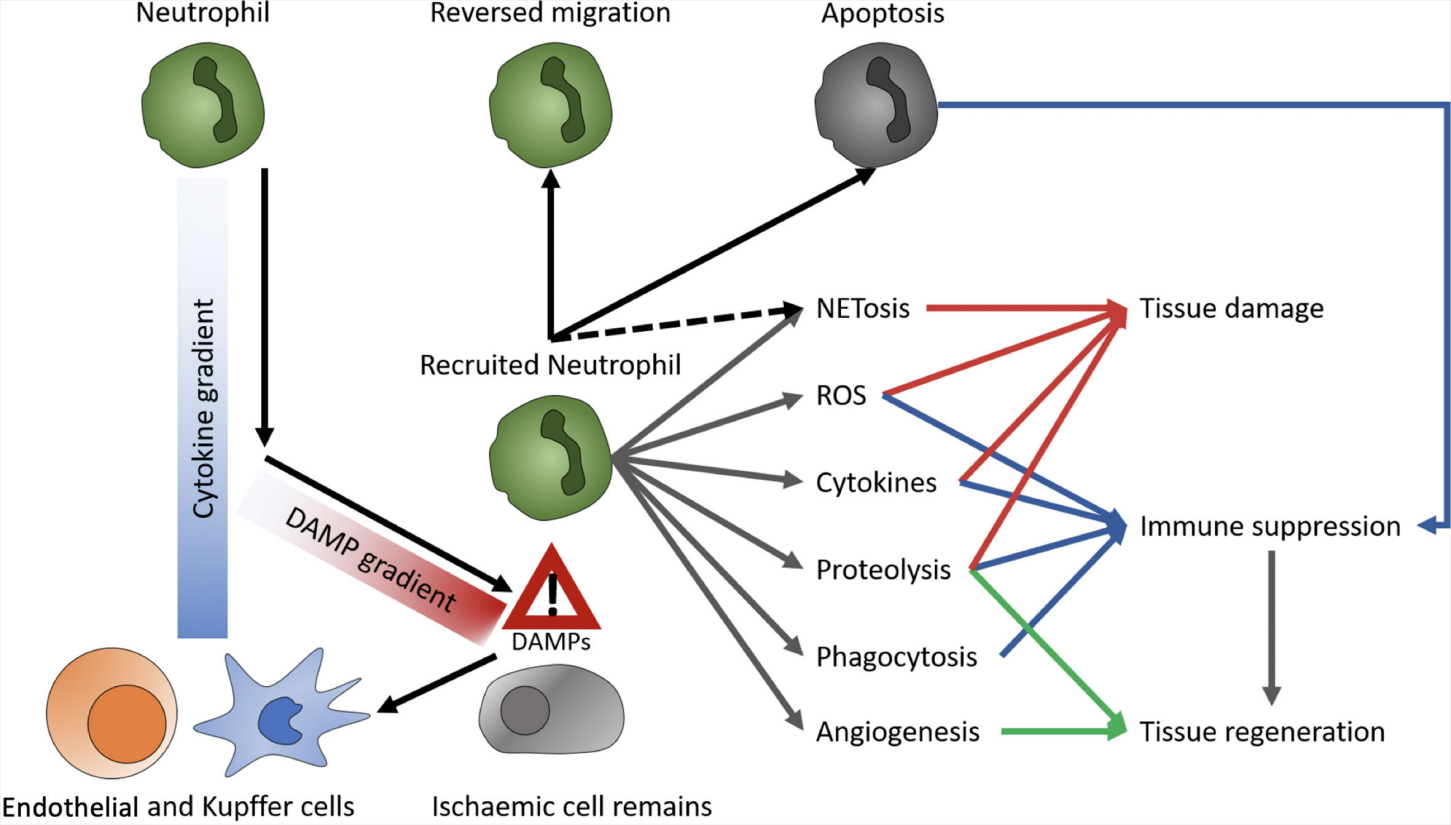
Role of neutrophils. DAMPs, danger- or death-associated molecular patterns; ROS, reactive oxygen species; NETs, neutrophil extracellular traps.

**Figure 3:**
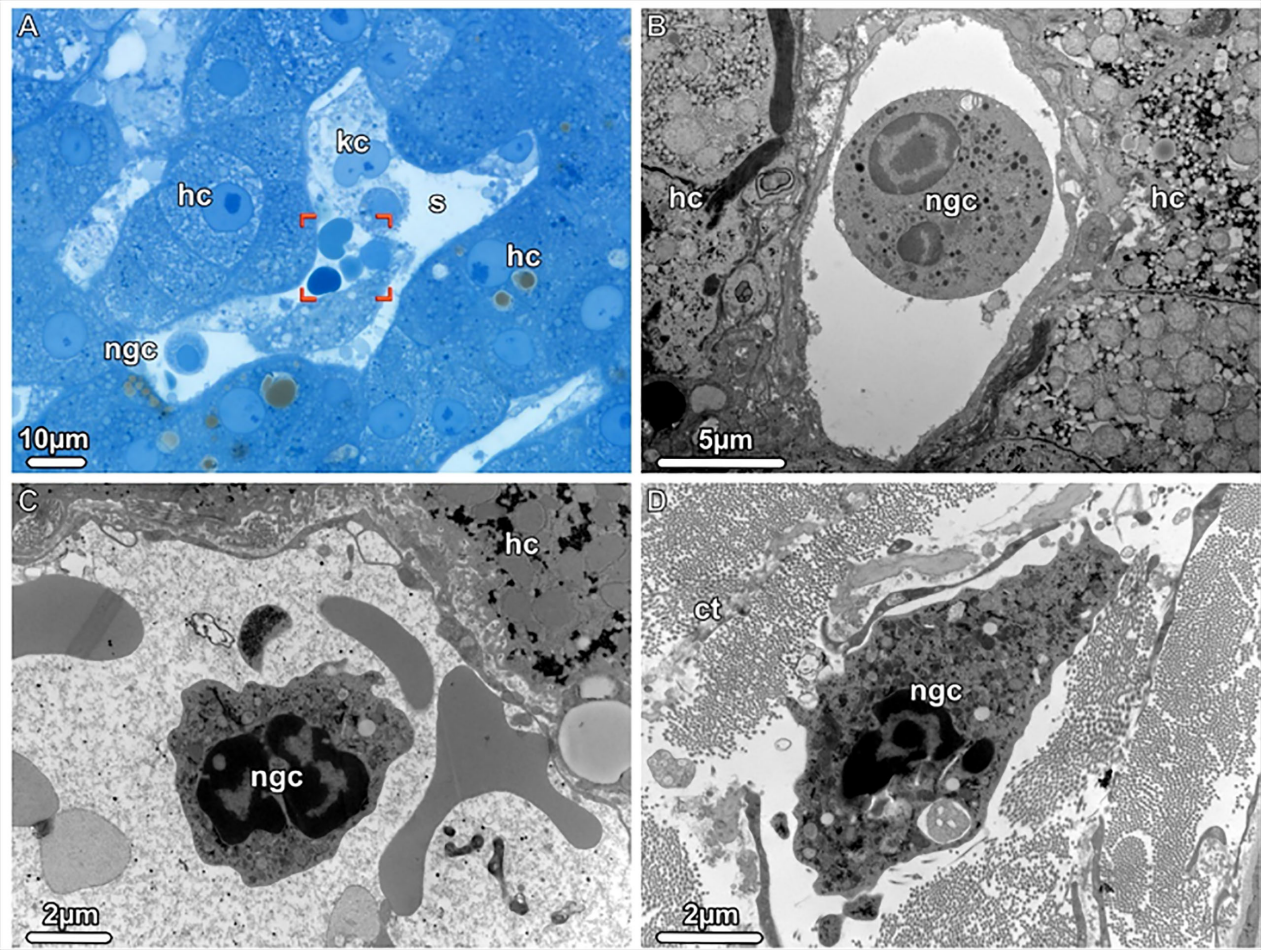
Neutrophil granulocytes detected during donor liver NMP, Light (LM) and Transmission electron microscopy (TEM). **(A)** LM. An overview of a liver sinusoid (s) containing Kupffer cells (kc), neutrophil granulocytes (gnc) and erythrocytes (brackets) is shown. **(B, C)** TEM. The sinusoids harbor mature as well as young neutrophilic granulocytes (ngc), both with a segmented nucleus. **(D)** TEM. A young neutrophil granulocyte (ngc) is seen in the dense connective tissue (ct) surrounding the liver, scattered between bundles of collagen fibrils. hc, hepatocytes.

Though initially considered a rather uniform, pro-inflammatory immune type, advances in analytical techniques suggest a variety of neutrophil subsets. The two most prominent neutrophil subtypes are N1 and N2. Their function is quite similar to their macrophage counterparts M1 and M2, resembling a pro-inflammatory and an anti-inflammatory, regenerative phenotype, respectively. *In vitro*, polarization of neutrophils toward an N1-like phenotype can be conducted with lipopolysaccharide (LPS), interferon gamma (IFNγ), and interferon beta (IFNβ). N2 cells differentiate upon treatment with L-lactate, adenosine, transforming growth factor beta (TGF-β), interleukin 10 (IL-10), prostaglandin E2 (PGE2), and granulocyte colony stimulating factor (G-CSF) (21). aged neutrophils tend to overactivation (22), whereas chronical exposure to pro-inflammatory conditions causes reduced inflammatory effector functions. These exhausted neutrophils, e.g., from patients with decompensated liver cirrhosis, reactivate their effector functions upon stimulation with toll like receptors (TLR) 7/8 and partially with TLR4 agonists (22, 23). Upon reperfusion of an organ, danger- or death-associated molecular patterns (DAMPs) released by ischemic tissue are detected by Kupffer cells (KC) and endothelial cells, creating a CXCL1/CXCL2 gradient to guide neutrophils chemotactically towards the site of injury. Moreover, KC secret IL1β, which induces the expression of intercellular adhesion molecule (ICAM)-1 in endothelial cells. In the liver, ICAM-1 enables binding of neutrophils to endothelia *via* MAC1 and subsequent transepithelial migration. When reaching the site of injury, neutrophils migrate towards DAMP signals, while disregarding the CXCL1/CXCL2 gradient (24, 25). Additionally, activation of the complement system has shown to promote neutrophil recruitment and subsequent tissue injury (26). The effects of complement on neutrophil migration in the context of liver MP are still unexplored, but might be relevant, as complement is produced in the liver itself and the organ is not perfused with whole blood ex situ. A recent study on human livers demonstrated that tissue neutrophil frequency significantly decreased at end of NMP, while no significant change was observed in the perfusate neutrophils (18). Thus, it was supposed that tissue neutrophils were activated and mobilized during NMP, based on a an augmented innate immune response triggered by reperfusion, which is known to cause excessive neutrophil influx to the liver from the vasculature (26). However, in liver NMP, the observed paradoxical decrease in donor-liver tissue neutrophils and the contemporanely constant perfusate neutrophil cell frequency may be explained by the continuous exposure of circulating neutrophils to non-endothelialized surfaces of the perfusion circuit, causing a proinflammatory state, resulting in adherence of neutrophils to circuits (2, 18). At the site of injury, neutrophils are involved in enhancing tissue damage and inflammation as well as tissue regeneration and immune suppression. As primary functions, neutrophils produce ROS and cytokines, perform formation of neutrophil extracellular traps (NET)osis, phagocytosis, proteolysis, and induce angiogenesis (**Figure 2**) (23, 24), which damage hepatocytes and enhance local inflammation. Delaying neutrophil exit in a Cathepsin-C (*Ctsc*) deficient mouse model also delayed revascularization of thermal liver injury (27). As NETs are extracellular structures, they act as DAMPs and consequentially enhance immune reactions (24, 28). In lung and kidney transplants, NETosis has been shown to be involved in graft rejection (24). Moreover, they promote coagulation and thereby disturb perfusion (24). Since, however, anti-coagulated blood is used for MP the significance of this process during *ex vivo* organ perfusion is questionable. On the other side, neutrophils participate in the clearance of debris by phagocytosis (24) **(** **Figure 3** **)**. Thereby, they reduce the amount of free DAMPs, which prevents subsequent inflammatory reactions. In alcoholic liver disease, neutrophils are mediators of liver damage, but they can diminish inflammation by clearing necrotic debris and induce hepatocyte regeneration *via* HGF (23). Similarly, antibody mediated depletion of neutrophils resulted in reduced clearance of debris as well as delayed vascularization and healing, in a model of thermal liver injury (29). These findings also highlight the role of neutrophils for tissue regeneration. Neutrophils participating in tissue revascularization, might display a targetable subset during MP (30). In a mouse model of pancreas transplantation, MMP9 turned out to be a key mediator for this process (27). This indicates that protein degradation is a key player not only in damaging hepatocytes, but also to induce revascularization and subsequent wound healing, additional to growth factor secretion. Further, neutrophils induce M2 polarization in macrophages. Targeting neutrophils is not common in LT or to prevent IRI. However, there have been efforts to reduce tissue damage by preventing neutrophil invasion. Inhibition of CXCL1 or CXCR2 diminished neutrophil migration and tissue damage. Besides, deactivation of matrix metalloprotease 9 (MMP9) demonstrated tissue protective effects in a mouse model of hepatic IRI, which has been attributed to impaired invasion (24, 26). On the other hand, MMP9 has been identified as a key player in revascularization (27). Hence, if MMP9 inhibition is beneficial for minimizing IRI in LT, this effect might be restricted to preventing neutrophil activity in the damaged tissue. As there is no neutrophil recruitment from the blood compartment during liver MP, it is questionable whether inhibition of neutrophil recruitment can show significant effects in an *ex vivo* liver perfusion setting. In analogy to preventing invasion, promoting neutrophil evasion into the perfusate might diminish immune mediated IRI. Both, intravascular and tissue resident neutrophils may be recruited into the blood flow (31). Additionally, neutrophils follow a chemotactic hierarchy, by which some migratory signals overwrite others (24). Hence, baiting neutrophils out of an organ prior to any in-tissue activities might diminish IRI. Alternatively, inducing reverse migration, for instance by treatment with LTB4 (30, 32), could reduce the number of locally active neutrophils.

In general, it appears that a total absence of neutrophils is not desirable to prevent IRI and tissue damage, also in MP. Instead, some activity is necessary for clearing debris, healing and regeneration. To provide a regenerative immune environment in general, *ex vivo* conditioning of donor livers and organs might be beneficial. Besides, elimination of excessive neutrophils may lead to reduction of liver injury and inflammation following LT (33).

## Kupffer cells are specialized hepatic antigen-presenting cells

**Figure 4:**
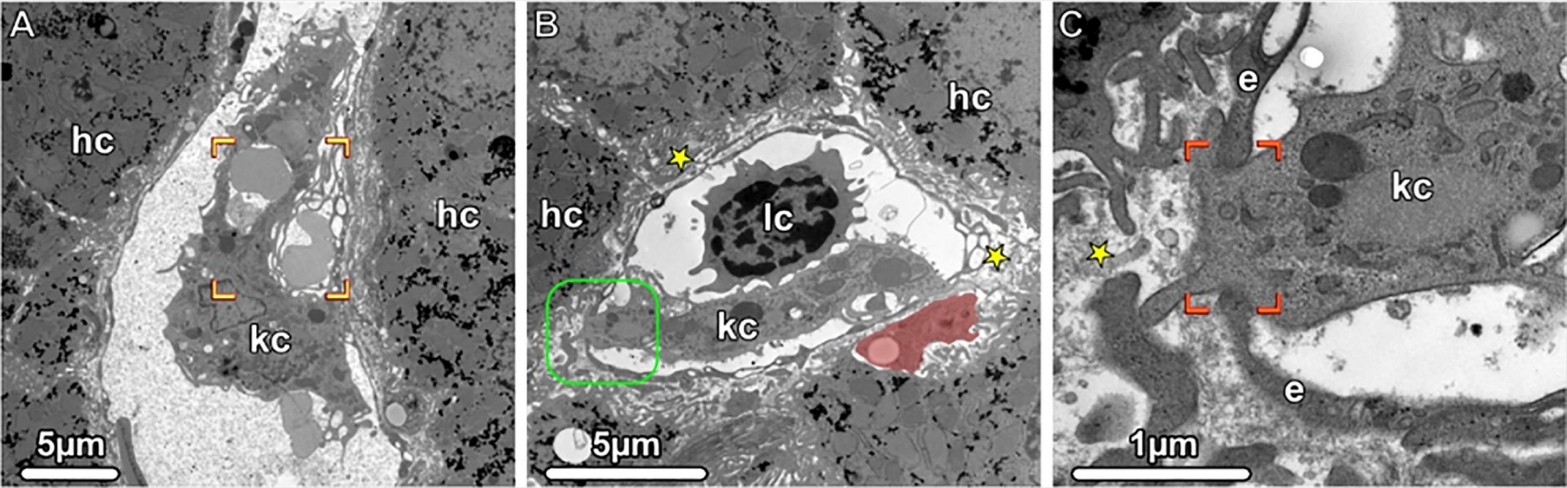
Kupffer Cells, Transmission electron microscopy (TEM). **(A, B)** Liver sinusoids with Kupffer cells (kc), lymphocytes (lc) and erythrocytes are shown. The damaged erythrocytes (brackets) are phagocytosed by a Kupffer cell, and in the Disse space (asterisks), an ito cell (highlighted in light red) is visible storing lipid droplets. The boxed area is shown in higher magnification in **(C)**. A portion of the Kupffer cell (kc) penetrates the fenestrated endothelium (e and brackets), thus gaining accesses to the Disse space (asterisks). hc, hepatocytes.

In 1876, von Kupffer identified liver-resident macrophages for the first time (34). These macrophages comprise about 90% of the total population of fixed macrophages in human body and form a third of the non-parenchymal liver cells (35). They are co-localized with sinusoidal endothelial cells, hepatic stellate cells and NK cells in the hepatic sinusoids (14) **(** **Figure 4** **)**. Depending on their distinct location, the function, morphology and number of KC changes (36). They have been described as the immunological sentinels of the liver and, depending on their surface marker phenotype or cellular functions, they are distinguished as having inflammatory as well as immunoregulatory properties.

The main role of KC is to clear the portal circulation from foreign materials and pathogens. In addition to their utility as antigen presenting cells (APCs), they are able to scavenge gut-derived pathogens, damaged erythrocytes and regulate iron and lipid metabolism (37). When doing so, KC release a battery pro-inflammatory cytokines such as IL-1, IL-6, IL-12, IL-18, tumor necrosis factor-alpha (TNF-α) and IFN-γ (14). With regard to LT, KC play a relevant mediating role in IRI, converting the liver into a highly inflammatory micromilieu and leading to PNF and EAD (3). During hepatic hypoxia, the resulting cellular stress triggers the release of endogenous DAMP molecules, which subsequently induces KC activation, release of cytokines and inflammatory mediators, in order to attract neutrophils and produce ROS (38). During IRI, the activation of TLR4 on KC enhances TNF secretion, which is further associated with hepatocyte apoptosis (39). Moreover, the activation of the complement system during IRI is responsible for KC-induced oxidative stress, triggering the formation of ROS and neutrophil recruitment to the ischemic liver (40). In the early state of hepatic IRI, KC produce inducible nitric oxide synthase (iNOS), which leads to reduced capillary perfusion and increased liver injury (41). On the other side, KC contribute to immune regulation, tissue repair and liver regeneration (42). After LT, KC act as APCs by increasing the expression of MHC class II and identifying recipient T cells migrating to the liver, which leads to T cell apoptosis and therefore play an important role during graft tolerance and survival (43, 44). In case of bacterial infection, KC produce anti-inflammatory cytokines such as IL-10, preventing activation of CD4+ T cells and limiting the adaptive immune response (45). Moreover, presentation of specific antigens by KC, induce IL-10 producing regulatory T cells, promoting antigen-specific tolerance. Contrarily, during acute liver injury, KC produce pro-inflammatory cytokines like IL-1, IL-6 and TNF-α as well as chemokines such as MIP-1α and RANTES (46). Human liver scRNAseq studies from three different groups have defined human KC facilitating the spatial mapping of these cells in human liver (47–49). Through identification of several markers, two distinct populations were distinguished, seeming to segregate into pro-inflammatory and immunoregulatory phenotypes. Specifically, MARCO (MAcrophage Receptor with COllagenous structure) is only expressed in non-inflammatory KC, while an inflammatory character was suggested by enriched expression of LYZ, CSTA and CD7454 (47).

Liver HOPE, which was shown to protect from IRI, downregulates activation of KC in a rat model (19). In contrast, increased levels of cytokines associated with KC activation (CCL-2, GM-CSF, IL-10, IFN-γ) were detected during human NMP together with the induction of an overall proinflammatory state (18). Based on the evidence that KC are key regulators of homeostasis, immune activation, tolerance induction (50), and that NMP triggers KC activation in human livers (18), targeting myeloid inflammation may help to improve organ function upon LT.

## Natural killer cells as early source of immunoregulatory cytokines

NK cells were initially described in 1975 based on their ability to kill tumor cells without prior sensitization (51). Following activation, NK cells offer a bridge between innate and adaptive immune system by augmenting early adaptive immune responses through the production of TNF-α and IFN-γ (52). The liver contains two NK cell subsets: conventional NK cells which circulate freely and liver resident NK cells (53). According to their surface markers, NK cells are divided into CD56bright and CD56dim subsets, where nearly to 90% are CD56dim, characterized by a high cytotoxicity. CD56bright NK cells, expressing a distinctive panel of chemokine receptors, are particularly enriched in the liver where they constitute over 50% of the total hepatic NK population, compared with 10–15% in peripheral blood [6]. They are located primarily in the sinusoids, produce a great amount of cytokines, but display low natural cytotoxicity (54, 55).

In the landscape of LT, NK cells have classically been described as proinflammatory, due to the increased expression of the activation marker CD69 and the natural cytotoxicity receptor NKp44, contributing to the release of inflammatory cytokines and cytolysis of donor cells. Hepatic NK cells express tumor necrosis factor-related apoptosis-inducing ligand (TRAIL), which is a potent inducer of hepatocyte cell death. The effect of TRAIL expression on NK cells during hepatic IRI was investigated and confirmed. Mice lacking TRAIL displayed significantly higher levels of liver injury and neutrophil infiltration (56). Additionally, it was assumed liver resident NK cells are responsible for the innate immune response in the early phase of IRI through self/non-self-recognition (57). Previous studies have demonstrated that viral infections induce NK cell accumulation and activation in the liver (58–60). Activated NK cells also work against biliary epithelial cells and contribute to hinder fibrosis through killing of hepatic stellate cells (61). A relative loss of a subpopulation of CD56+CD16− NK cells was observed in fibrotic human liver tissue using scRNAseq [24]. The interaction between liver NK cells and KC might trigger the production of IFN-γ and TNF-α, contributing to the development of fulminant hepatitis [87]. Furthermore, TRAIL+ NK cells could eliminate immature DC (62) thereby impact the advancing of certain liver diseases.

There is also evidence that NK cell populations have important immunoregulatory functions (58). A high proportion of hepatic NK cells express the inhibitory receptor NKG2A. In contrast to their peripheral blood counterparts, they are capable of twice the cytotoxicity level, resulting in depleted activation of T cells and tolerance induction after LT (63). During hepatitis C virus (HCV) infection, they can inhibit DC activation by producing the suppressive factors transforming growth factor-beta (TGF-ß) and IL-10. Subsequently, the resulting tolerogenic DC trigger the expansion of regulatory T cells, contributing to the induction of an immunotolerant state (64). Diverse studies have investigated the role of NK cells during graft rejection (14, 65). The absence of recipient-derived NK cells or the decrease in IFN-γ production after LT has been shown to be advantageous during both allograft rejection and tolerance induction in a rat model (66). In addition, 13 genes that are highly expressed in NK cells were found to be present in LT recipients with graft tolerance, which further indicates/provides further evidence that NK cells are involved in tolerance induction (67). The conflicting role of NK cells is still not fully understood; however, it is likely that NK cells play a role in the development of tolerance, thus providing a novel rationale for minimizing immunosuppression in recipients of livers with greater proportions of NK cells (52). A fist analysis on human liver NMP, showed that the composition of leukocytes within the perfusate after organ procurement and cold flush consisted mainly of neutrophils (about 55%) and NK cells (about 13%). While no significant changes regarding the NK cell compartments within the tissue were observed, duration of NMP was associated with significant decreases in frequencies of NK cells when serial perfusate was analyzed (18). In the light of the growing use of MP in routine LT, future studies focusing on immune interactions at the time of LT and during rejection episodes, combined with cell dynamics during MP, should clarify the role of NK cells in rejection and tolerance.

## Dendritic cells are key players in induction and regulation of immune responses

In 1973 Cohn and Steinman discovered a specific type of immune cell, the dendritic cell (DC) (68), which plays an important role as sentinel of the immune system, as they are deployed throughout the body and monitor their surroundings for antigens and danger signals derived from pathogens or tissue damage. DC can be categorized into two separate lineages: conventional/myeloid DC (mDC) are specialized APCs capable of beginning and driving specific T cell immune responses, whereas plasmacytoid DC (pDC) are able to rapidly produce type 1 interferons and regulate inflammatory responses (12). All populations of DC have now been identified in human liver tissue using flow cytometry (69) and scRNAseq, with markers such as LILRA4, XCR1 and CD1c in order to distinguish them (12). Human mDC express high levels of CD11c and are classified according to their expression of CD1a (also known as blood dendritic cell antigen [BDCA] 1) versus CD141 (also known as BDCA3) (70). The BDCA1+ DC differentiate under the influence of interferon regulatory factor 4 (IRF4), express high levels of CD1b, CD14, and SIRP-α and promote T helper (Th) 2 responses. BDCA3+ DC develop under IRF8, express XCR1, CLEC9F, BTLA4 and secrete IL12-promoting Th1 responses in CD4+ T cells (71). In the healthy liver 70% are BDCA1+, while 30% are BDCA3+ (69). On the other side, pDC display an accentuate response to viral pathogen associated molecular patterns (PAMPs) and the synthetize IFNα (72). They express CD123, CD14 and CD303 (also known as BDCA2), besides initiating antiviral immune response and secreting IFNα/β (72, 73).

In the healthy liver, DC are mainly immature cells, capable to capture and process antigens (12). In the context of LT, mDC and pDC have been explored as key players of graft rejection and immune tolerance. This was also observed in a transgenic murine model, in which depletion of DC resulted in loss of liver tolerance and allograft rejection (74). Simultaneously, DC can also promote liver graft rejection, according to studies where donors were treated with Fms-like tyrosine kinase receptor 3 (Flt3)-ligand, causing not only increased DC numbers, but also augmented CD80 and CD86 expression (75, 76). Although mDC can induce graft rejection, their baseline state is likely to promote liver tolerance, as a consequence of several mechanisms driving a close interaction with hepatic stellate cells (HSCs) (72). There is less evidence concerning the role of liver DC in IRI, but it was shown that this innate immune pathology follows very different rules from the T cell tolerance experienced in LT. In this context, pDC appear to be key players of the immunopathology. Their expression of TLR4 and TLR9 renders them highly responsive to DAMPs released by ischemia-injured cells, and their response is to secrete IFNα, IL6, and TNF-α, which further augment tissue injury. Contrarily, mDC suppress IRI, through the secretion of anti-inflammatory cytokines (72). Future research should examine how these populations differ functionally in regulating hepatic immunity, how they contribute to liver disease development (37) and which role they may assume in the context of liver MP. Dendritic cell–derived extracellular vesicles (DC-EVs) have emerged as a novel immunomodulatory agent in LT. DC therapies are able to induce a tolerogenic immune environment through secretion of anti-inflammatory cytokines and induction of T cell anergy, nearly to attenuating hypoxic injury and promoting allograft survival (77–79). The administration of EVs directly to the liver during NMP may guarantee their targeted delivery, providing time for modulation of the immune environment prior to LT and maximizing the therapeutic potential (77, 80).

## The liver adaptive immune system: the specific role of lymphocyte subtypes in hepatic immune activation

The highly specialized liver adaptive immunity, consisting of humoral and cellular immunity, is able to provide long-term protection with immunological memory, while promoting self-tolerance (12). The liver possess an immunosuppressive microenvironment, which means that hepatic adaptive immune cells become readily tolerogenic, endorsing the death of effector cells and the “education” of regulatory cells (81).

Based on different functions and phenotypes, the most relevant T lymphocytes implicated in adaptive immunity include CD4 T cells and CD8 T cells, additionally characterized into several subgroups. CD4 T cells counted various functional categories. While helper T (Th)1, Th2, Th17 and follicular helper T (Tfh) cells, mostly support innate and adaptive immune responses, the regulatory T (Treg) cells (CD4+, CD25high, CD127low, FoxP3+), usually overrule the augmented inflammatory reaction resulting from innate and adaptive immunity and restore immune homeostasis. Multiple immunosuppressive mechanisms have been attributed to Tregs such as the secretion of anti-inflammatory cytokines, depletion of crucial growth factors, and direct cytotoxic killing of effector cells (13). The liver immune response is mostly associated with a strong CD4 and CD8 T cell reaction. CD8 T cells play a key role in this context, because they recognize peptides from intracellular pathogens in the context of MHC I. Subsequently they initiate diverse effector mechanisms, including the production of cytokines, such as IFN-γ and TNF-α, and further cytolytic mechanisms, by releasing granule contents like perforin and granzyme and by triggering Fas-mediated apoptosis (12, 82). Jassem et al. observed reduced numbers of proinflammatory cytokines IFN-γ and IL-17 producing CD4 and CD8 T cells on human livers subjected to NMP (2).

A main characteristic of the adaptive immune system is to form a pool of memory T cells, enabling an effective immune response after pathogen re-exposition. The liver displays intrahepatic tissue resident memory (TRM) cells, which require a different cytokine milieu and have diverse phenotypes compared to their counterparts in the blood (83, 84). While in mice the non-circulating liver TRM account for 40–60% of the liver-resident T cells, this amount is significantly higher in humans, where it ranges between 60 and 80%. In order to infiltrate the liver, memory T cells express liver-specific homing markers like CD103, LFA-1, CXCR6 or CXCR3. However, TRM have the ability to return back into the bloodstream by upregulating CCR7 and S1PR1 (83). After activation, CD8+ TRM cells produce TNF-α and IFN-γ, acquiring the ability to directly lyse target cells. The proinflammatory cytokine expression on TRM cells is elevated in comparison to the circulating memory T cells, denoting an efficient effector function at the tissue-site of infection. Additionally, CD8 TRM cells recruit other immune cells by chemokine production after antigen recognition (85). In comparison to CD8 TRM cells, the CD4 TRM cell amount in the human liver is low, potentially due to a reversed CD8/CD4 ratio compared to the blood (86). T cells are highly involved in the pathogenesis of IRI, which includes not only CD4 T cells, but also CD8 and γδ T cells. Recently, it was demonstrated that NMP significantly increased the proportion of T cells in the perfusate throughout the course of perfusion. This may suggest that donor tissue T cells are mobilized into the perfusate during NMP (18). However, tissue T cell frequency remains mostly unchanged throughout the course of NMP. It was suggested that perfusate T cells permanently migrate back into the liver tissue, generating a dynamic T cell trafficking loop between perfusate and tissue compartments (18).

B cells have been considered a main component of the adaptive immune response, also contributing to mediate graft injury. They comprise about 5% of the liver lymphocytes. While immature, chronically activated B cells are effective APCs, thought to augment T cell-mediated rejection, mature, late lineage B cells produce donor-specific antibodies and contribute to both acute and chronic allograft injury (87). B cells additionally produce cytokines and chemokines modulating the extent of the alloimmune response. As described for T cells, also B cells should also be capable of both augmenting and suppressing immune responses (88). B cell dysfunction has been implicated in the pathogenesis of numerous immune mediated liver diseases, such as autoimmune hepatitis (AIH), IgG4-related hepatobiliary disease (IgG4-HBD), primary biliary cholangitis (PBC) and primary sclerosing cholangitis (PSC) (87, 89, 90). The role of regulatory B cells (Breg) has been established in the context of autoimmunity (88), however, the lack of molecular markers is still a limiting factor for their further characterization. They are often identified by the production of IL-10. Moreover, there is evidence that Breg and Treg may collaborate in order to promote tolerance, through the mediating effect of IL-10 (88). In the context of liver NMP, continuously increasing frequencies of B cells were detectable within the perfusate over the entire NMP course (18). The authors concluded that this could probably be explained by the controlled oxygenated rewarming after substantial cold ischemia time. Likewise, NMP with controlled oxygenated rewarming of liver after cold storage resulted in significantly improved recovery upon reperfusion associated with cold-stored only grafts (91).

## Perspective and conclusion

To date there is conflicting data whether MP exerts a pro- or an anti-inflammatory effect on donor livers prior to LT. While there is evidence that a great amount of leukocytes is mobilized into the perfusate during liver MP and an increase in proinflammatory cytokines are found with prolonged perfusion in some studies, also upregulation of regenerative pathways and primarily anti-inflammatory mediators in the course of human liver NMP are observed. Previously, refining the perfusate composition with anti-inflammatory agents, as prostaglandin E1, antiplatelet and fibrinolytic factors during *ex vivo* warm liver MP improved the outcome after LT in a pig model (92). Further, liver-resident immune cells gained an activated phenotype during NMP on gene and protein levels in a rat model, which could be reduced through therapeutic intervention with anti-inflammatory IL-10 and TGF-β (93). Moreover, a time-dependent increase in DAMPs levels and inflammatory cytokines during MP, particularly pronounced at higher preservation temperatures, was shown in another rat model (94). In the past, differential centrifugation, sedimentation, cell washing, freezing and thawing, and filtration have been used to leukodeplete the perfusate used for MP (95). The efficacy of leukocyte depletion filters (LDFs) was previously evaluated in the context of normothermic *ex vivo* lung perfusion (96) and in LT for oncological disease, where some types of LDFs could reduce the risk for reintroducing tumor cells (97). While in human organ perfusion, leukocyte-depleted packed red blood cells are immediately available from blood banks, controlling white blood cells-mediated damage, the use of whole blood-based perfusate still remains a limiting factor in experimental animal models of MP (98). Active mobilization and elimination of hepatic immune cells using leukocyte depletion filters (LDFs) during liver MP is an option and may seem reasonable as this reduces the antigenic load of the organ, hence diminishing acute rejection after LT. On the other hand, specific subtypes of immune cells have been shown to be critically involved in regenerative, healing and tolerogenic processes and hence indiscriminate withdrawal may be contra productive. However, how much elimination is needed to balance destructive versus regenerative processes in the liver while on the perfusion device? In this context it might be advisable to specifically and actively promote migration and trafficking of highly proinflammatory immune cells by strongly activating the inflammatory cascade. The high levels of proinflammatory cytokines could then be filtered from the perfusate together with the correspondingly acting cells. Future investigations should consider the possible application of leucocyte filtering during MP as therapeutic strategy.

With the development of prolonged organ perfusion, and the possibility of the MP systems to add substances and therapeutics directly into the perfusate which then circulate directly through the liver, it may also be an option to administer factors affecting the maturation state of immune cells or inducing a regulatory and/or regenerative phenotype. Moreover, *ex vivo* expanded subtypes of immune cells exerting an advantageous effect during liver MP may be administered into the perfusate for therapeutic purposes in the future.

To take the next steps it is of uttermost importance to elucidate and understand (i) the role of hepatic immune cells during MP (ii) how MP influences the immune cell repertoire, and (iii) how this affects the immune microenvironment and milieu. This should lay the groundwork for active immune modulation and induction of regeneration during liver MP as a future goal.

## Author contributions

Designed and outlined the review: FM, SaS, HT; designed the figures: FM, SaS, MA, GH, BM; drafted the review: FM, SaS, GH, MA, HT; critically revised review and approved final version: all.

## Acknowledgments

We thank all members of the NMP team at the Department of Visceral, Transplant and Thoracic Surgery as well as the organLife team.

## Conflict of interest

The authors declare that the research was conducted in the absence of any commercial or financial relationships that could be construed as a potential conflict of interest.

## Publisher’s note

All claims expressed in this article are solely those of the authors and do not necessarily represent those of their affiliated organizations, or those of the publisher, the editors and the reviewers. Any product that may be evaluated in this article, or claim that may be made by its manufacturer, is not guaranteed or endorsed by the publisher.
